# Multivariate and subgroup analyses of a randomized, multinational, phase 3 trial of decitabine vs treatment choice of supportive care or cytarabine in older patients with newly diagnosed acute myeloid leukemia and poor- or intermediate-risk cytogenetics

**DOI:** 10.1186/1471-2407-14-69

**Published:** 2014-02-06

**Authors:** Jiří Mayer, Christopher Arthur, Jacques Delaunay, Grzegorz Mazur, Xavier G Thomas, Agnieszka Wierzbowska, Farhad Ravandi, Erhan Berrak, Mark Jones, Yuhan Li, Hagop M Kantarjian

**Affiliations:** 1 Masaryk University Hospital Brno and Central European Institute of Technology (CEITEC), Masaryk University, Brno, Czech Republic; 2 Royal North Shore Hospital, St. Leonards, New South Wales, Australia; 3 Nantes University Hospital, Nantes, France; 4 Wroclaw Medical University, Wroclaw, Poland; 5 Affiliation at time of study: Hôpital Edouard Herriot, Lyon, France; 6 Medical University of Lodz, Lodz, Poland; 7 The University of Texas MD Anderson Cancer Center, Houston, TX, USA; 8 Eisai Inc., Woodcliff Lake, NJ, USA; 9 Current address: Hôpital Lyon-Sud, Lyon, France

**Keywords:** Decitabine, Acute Myelocytic Leukemia, Elderly, Treatment

## Abstract

**Background:**

Compared with younger patients, older adults with acute myeloid leukemia (AML) generally have poorer survival outcomes and less benefit from clinical trials. A recent phase 3 trial demonstrated a trend toward improved overall survival (OS) with decitabine, a hypomethylating agent, compared with treatment choice of either cytarabine or supportive care (7.7 months, 95% CI: 6.2–9.2 vs 5.0 months, 95% CI: 4.3–6.3, respectively) in older adults with newly diagnosed AML. The current analyses investigated prognostic factors for outcomes in this trial and examined OS and responses in prespecified subgroups.

**Methods:**

A multivariate Cox proportional hazards model was used to investigate effects of demographic and baseline characteristics, including age, sex, cytogenetic risk, AML type, ECOG Performance Status, geographic region, bone marrow blasts, platelets, and white blood cells on OS, based on mature data. Similar analyses were conducted with a logistic regression model to predict response rates. Prespecified subgroup analyses were performed for OS and response rates, also using mature data.

**Results:**

Patient characteristics that appeared to negatively influence OS included more advanced age (hazard ratio [HR] 1.560 for ≥75 vs <70 years; p = 0.0010), poorer performance status at baseline (HR 0.771 for 0 or 1 vs 2; p = 0.0321), poor cytogenetics (HR 0.699 for intermediate vs poor; p = 0.0010), higher bone marrow blast counts (HR 1.355 for >50% vs ≤50%; p = 0.0045), low baseline platelet counts (HR 0.775 for each additional 100 × 10^9^/L; p = 0.0015), and high white blood cell counts (HR 1.256 for each additional 25 × 10^9^/L; p = 0.0151). Regarding geographic regions, patients from Western Europe had the longest median OS. Response rates favored decitabine for all subgroups investigated, including patients ≥75 years (odds ratio 5.94, p = 0.0006).

**Conclusion:**

Response to decitabine in AML is associated with known prognostic factors related to both patient demographics and disease characteristics.

**Trial registration:**

ClinicalTrials.gov identifier NCT00260832

## Background

The European LeukemiaNet [1], European Society of Medical Oncology [2], and National Comprehensive Cancer Network (US) [3] recently indicated that management of patients aged ≥60 years with acute myeloid leukemia (AML) should be guided by performance status and presence of comorbidities. Until recently, treatment approaches had proven difficult in older patients. However, international treatment guidelines for AML now include low-intensity cytarabine, 5-azacytidine, and decitabine as therapeutic options [1,3].

Decitabine, a hypomethylating agent, was approved by the European Medicines Agency in late 2012 for treatment of patients aged ≥65 years with newly diagnosed de novo or secondary AML [4]. Decitabine appears to have direct cytotoxic effects and is believed to affect cellular differentiation and apoptosis. In the US, it is indicated for treatment of previously treated and untreated de novo and secondary myelodysplastic syndrome (MDS) of all French-American-British subtypes and intermediate-1, intermediate-2, and high-risk International Prognostic Scoring System groups [5]. In patients with MDS [6], chronic myelomonocytic leukemia [6], or AML, intravenous (IV) decitabine 20 mg/m^2^ administered daily for 5 consecutive days every 4 weeks was well tolerated [7].

A recently reported phase 3 trial in older patients (≥65 years) with newly diagnosed AML and poor- or intermediate-risk cytogenetics compared the efficacy and safety of decitabine with patient’s treatment choice (TC), upon physician’s advice, of supportive care (SC) or cytarabine [8]. The study had a planned clinical cutoff date of October 28, 2009; 396 deaths had occurred. The primary efficacy analysis (October 2009) showed a nonsignificant trend toward increased median overall survival (OS) with decitabine (7.7 months; 95% confidence interval [CI]: 6.2–9.2 months) versus TC (5.0 months; 95% CI: 4.3–6.3 months), with an estimated hazard ratio (HR) of 0.85 (p = 0.108). In a post hoc analysis using mature data (clinical cutoff October 29, 2010) [8], 446 deaths had occurred. Median OS was the same as that in the 2009 analysis, but with improved HR (0.82; 95% CI: 0.68–0.99; nominal p = 0.037) with decitabine. At the 2009 cutoff, significantly improved remission rates were observed with decitabine versus TC (complete response [CR] or CR with incomplete platelet recovery [CRp], 17.8% vs 7.8%, respectively; p = 0.001), and decitabine was well tolerated.

Using mature (2010) data from the phase 3 trial [8], this multivariate analysis aimed to identify potential predictors of survival and response in this older population with AML. Prespecified analyses of response and survival by subgroups are also reported.

## Methods

### Patients and study design

Patients aged ≥65 years with newly diagnosed, histologically confirmed de novo or secondary AML (≥20% blasts), poor- or intermediate-risk cytogenetics (Southwest Oncology Group categorization [9]), and Eastern Cooperative Oncology Group (ECOG) Performance Status (PS) of 0–2 were eligible. Excluded patients had t(15;17), t(8;21), or inv(16) karyotype abnormalities. Complete protocol details were previously reported [8].

This study, conducted in 15 countries, was approved by institutional review boards or independent ethics committees and conducted in accordance with the Declaration of Helsinki. All patients provided written, informed consent.

Patients in this randomized, open-label, phase 3 trial indicated, with physician’s advice, their preferred TC of SC or once-daily cytarabine 20 mg/m^2^ subcutaneously for 10 consecutive days every 4 weeks. Patients were randomized 1:1 to receive decitabine or TC, stratified by age, cytogenetic risk, and ECOG PS. Once-daily decitabine 20 mg/m^2^ was administered as a 1-hour IV infusion for 5 consecutive days every 4 weeks. Treatment continued until relapse or progressive disease (PD), death, unacceptable toxicity, lack of clinical benefit, intercurrent illness preventing treatment, or patient/physician request.

The primary objective was to compare OS (from randomization to death) in patients receiving decitabine versus TC. Secondary objectives were to compare response rates, including CR and CRp, based on bone marrow biopsies and aspirates. Patients were followed monthly for 2 years post-randomization, then every 2 months for 3 years for OS and PD until death or loss to follow-up.

### Statistical analysis

The primary OS analysis was based on the 2009 clinical cutoff date [8]. To provide additional data, a post hoc analysis evaluated mature 2010 survival data [8]. Mature data were utilized for this multivariate analysis. At the 2010 clinical cutoff, >90% of events were recorded.

A multivariate Cox proportional hazards model was used to investigate the effects of the following baseline characteristics on OS: gender, age (<70, 70 to <75, and ≥75 years), baseline cytogenetic risk (intermediate vs poor), AML type (de novo vs secondary), baseline ECOG PS (0/1 vs 2), region (North America/Australia, Western Europe, Eastern Europe, or Asia), baseline bone marrow blasts (>50% vs ≤50%), baseline platelet counts (at each unit of 100 ×10^9^/L), and baseline white blood cell (WBC) counts (at each unit of 25 ×10^9^/L). These potential prognostic factors were evaluated all at once and p values determined without adjustment for multiple testing.

For probability of achieving CR or CRp, a logistic regression model was used to investigate effects of the same demographic and baseline characteristics. Again, these factors were evaluated simultaneously and p values determined without adjustment for multiple testing.

Prespecified subgroup analyses were additionally performed based on mature data to compare OS between decitabine and TC using the Cox regression model stratified by age, cytogenetic risk, and ECOG PS (same model used for the study’s primary OS analysis) [8]. Subgroups included those with baseline bone marrow blasts ≥20% and ≤30% versus >30%; de novo versus secondary AML; intermediate-risk versus poor-risk cytogenetics; ages <70, 70–74, and ≥75 years; and ECOG PS of 0–1 versus 2. Regional subgroups were also compared. The subanalysis for response (CR + CRp) was performed in the same subgroups, using 2-sided Fisher’s exact test (same method used for the study’s secondary responder analysis) [8].

The phase 3 study was registered with ClinicalTrials.gov with identifier NCT00260832.

## Results

### Patients

**Figure 1:**
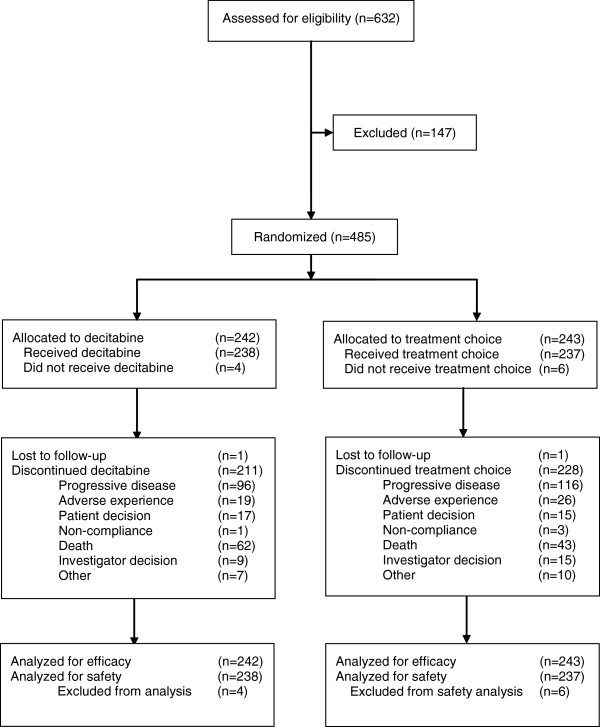
**Flow diagram for phase 3 study.** Reprinted with permission from Kantarjian H, et al. *J Clin Oncol* 2012 [8].

Between January 2006 and April 2009, 485 patients were randomized at 65 sites. The efficacy population comprised 242 decitabine recipients and 243 TC recipients (cytarabine, n = 215; SC, n = 28; Figure 1). Baseline clinical characteristics and patient demographics were similar between treatment groups (Table 1) and reflected a high-risk population [8]. Approximately 71% of patients were aged ≥70 years, with secondary AML in 35.3%, poor cytogenetic risk AML in 36.0%, and ECOG PS of 2 in 25.8% of patients; median baseline bone marrow blasts were 46.0%.

**Table 1:** **Patient demographics and baseline clinical characteristics**[8]

**Parameter**	**Total TC (n = 243)**	**Decitabine (n = 242)**
Median age, years (range)	73.0 (64.0–91.0)	73.0 (64.0–89.0)
<70 years	70 (28.8)	71 (29.3)
70–74 years	74 (30.5)	76 (31.4)
≥75 years	99 (40.8)	95 (39.3)
Sex, n (%)		
Female	92 (37.9)	105 (43.4)
Male	151 (62.1)	137 (56.6)
Median BSA, m^2^ (range)	1.80 (1.3–2.7)	1.82 (1.4–2.6)
Median time since AML diagnosis, days (range)	15.0 (0–398.0)	14.0 (3.0–346.0)
Type of AML, n (%)		
De novo	157 (64.6)	155 (64.0)
Secondary	84 (34.6)	87 (36.0)
Bone marrow blasts, n (%)		
<20%	8 (3.3)	4 (1.7)
20%–30%	58 (24.1)	65 (27.0)
>30%–50%	74 (30.7)	67 (27.8)
>50%	101 (41.9)	105 (43.7)
ECOG PS, n (%)		
0 or 1	178 (73.2)	182 (75.3)
2	65 (26.7)	60 (24.8)
Cytogenetics, n (%)		
Intermediate risk	154 (63.6)	152 (63.1)
Poor risk	87 (36.0)	87 (36.1)
Median hemoglobin, g/dL (range)	9.4 (5.0–12.6)	9.3 (5.2–15.0)
Platelets, n	213	225
Median, 10^9^/L (range)	50.00 (6.0–490.0)	58.00 (6.0–487.0)
White blood cells, n	236	237
Median, 10^9^/L (range)	3.69 (0.5–80.9)	3.10 (0.3–127.0)

AML, acute myeloid leukemia; ECOG PS, Eastern Cooperative Oncology Group Performance Status; TC, treatment choice, with physician’s advice.

Considering enrollment by region and country (Table 2), the largest proportion of patients in each treatment group was from Eastern Europe (46.5%, TC; 45.0%, decitabine).

**Table 2:** **Trial enrollment by region and country**[8]

**Region**	**Country**	**TC (n = 243)**	**Decitabine (n = 242)**
		**Patients, n (%)**	**Patients, n (%)**
**Eastern Europe**		113 (46.5)	109 (45.0)
	Poland	47 (19.3)	41 (16.9)
	Russian Federation	29 (11.9)	28 (11.6)
	Czech Republic	23 (9.5)	24 (9.9)
	Serbia	10 (4.1)	12 (5.0)
	Hungary	3 (1.2)	4 (1.7)
	Romania	1 (0.4)	0 (0.0)
**North America/ Australia**		69 (28.4)	51 (21.1)
	United States	27 (11.1)	21 (8.7)
	Australia	22 (9.1)	16 (6.6)
	Canada	20 (8.2)	14 (5.8)
**Western Europe**		34 (14.0)	51 (21.1)
	France	24 (9.9)	31 (12.8)
	Spain	10 (4.1)	20 (8.3)
**Asia**		27 (11.1)	31 (12.8)
	Taiwan	27 (11.1)	31 (12.8)

Percentages were calculated using the number of patients in each group as the denominator.

SC, supportive care; TC, patient's choice of treatment with physician's advice.

### Multivariate analysis

After adjustment for potential prognostic factors, the HR for treatment effect on OS based on mature data was 0.799 (95% CI: 0.653–0.978), favoring decitabine (Table 3). Patient characteristics appearing to negatively influence OS at the 0.05 level included advanced age, poorer baseline ECOG PS, poor cytogenetic risk, higher bone marrow blast counts, low baseline platelet counts, and higher WBC counts. Geographically, being in the Western European subgroup was predictive of a longer median OS.

**Table 3:** Overall survival: multivariate proportional hazard analysis

**Variable**	**Hazard ratio**	**95% CI**	**P value**
Treatment: decitabine vs TC	0.799	(0.653–0.978)	0.0296
Sex: male vs female	1.125	(0.912–1.388)	0.2703
Age group			
70–74 vs <70 years	1.311	(1.004–1.711)	0.0468
≥75 vs <70 years	1.560	(1.198–2.032)	0.0010
Baseline cytogenetic risk: intermediate vs poor	0.699	(0.565–0.865)	0.0010
Type of AML: de novo vs secondary	1.110	(0.893–1.380)	0.3452
Baseline ECOG PS: 0 or 1 vs 2	0.771	(0.607–0.978)	0.0321
Geographic region [8]			
Eastern Europe vs North America/Australia	1.118	(0.849–1.473)	0.4263
Western Europe vs North America/Australia	0.727	(0.523–1.010)	0.0572
Asia vs North America/Australia	1.047	(0.728–1.505)	0.8052
Western Europe vs Eastern Europe	0.650	(0.482–0.877)	0.0048
Western Europe vs Asia	0.694	(0.472–1.021)	0.0637
Baseline bone marrow blast: >50% vs ≤50%	1.355	(1.099–1.672)	0.0045
Baseline platelets (10^9^/L)^a^	0.775	(0.663–0.907)	0.0015
Baseline WBC (10^9^/L)^b^	1.256	(1.045–1.509)	0.0151

AML, acute myeloid leukemia; CI, confidence interval; ECOG PS, Eastern Cooperative Oncology Group Performance Status; TC, patient's choice of treatment with physician's advice; WBC, white blood cell counts.

^a^Hazard ratio less than 1 indicates lower risk associated with each additional 100 × 10^9^/L in baseline platelet counts.

^b^Hazard ratio greater than 1 indicates higher risk associated with each additional 25 × 10^9^/L in baseline WBC counts.

After adjustment for the same set of potentially prognostic factors in the logistic regression analysis for response (CR + CRp), an advantage was noted for decitabine over TC in response rate (odds ratio [OR] 2.751 [95% CI: 1.487–5.091; p = 0.001). The only patient characteristic significantly influencing response rate was sex (male vs female, OR 0.551 [95% CI: 0.304–0.998]; p = 0.049).

### Subgroup analyses

Prespecified analyses of subgroups were performed for OS and response (CR + CRp) using 2010 mature data. In general, OS results were consistent with those using 2009 data [8], favoring treatment with decitabine with 3 exceptions. In the mature data set, the treatment effect on OS in the Western European subgroup favored TC, which was markedly different from the treatment effect seen in the overall population, which favored decitabine. Similarly, in the subgroup of patients with baseline bone marrow blasts of 20% to 30%, the treatment effect on OS favored TC, but favored decitabine in the overall population. Finally, for patients aged <70 years, HR was close to unity, with neither treatment favored. Overall survival data for these subsets were reported in detail elsewhere [8].

**Figure 2:**
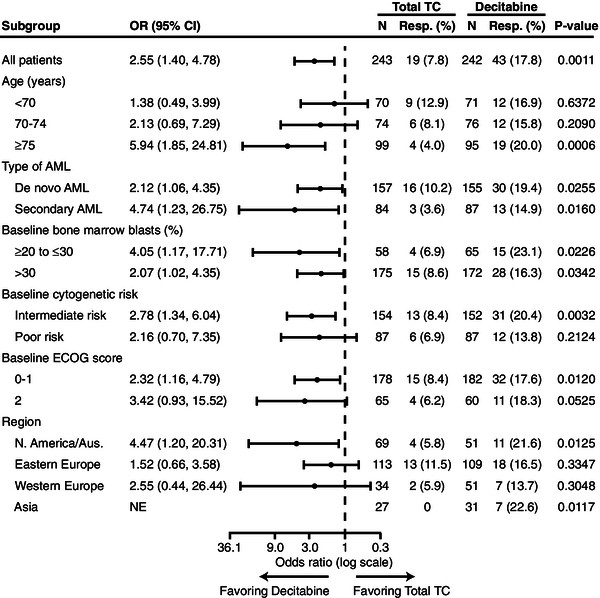
**Subanalyses of patients with response to either decitabine or treatment choice (complete remission or complete remission with incomplete platelet recovery).** P values were based on 2-sided Fisher’s exact test and stratified by age, cytogenetic risk, and Eastern Cooperative Oncology Group Performance Status. AML, acute myeloid leukemia; Aus., Australia; CI, confidence interval; ECOG, Eastern Cooperative Oncology Group; HR, hazard ratio; Med, median (months); NE, not estimable; OR, odds ratio; Resp, responder; TC, patient’s choice of treatment with physician’s advice.

In the analysis of response rates, all subgroups favored decitabine (Figure 2).

#### Age

In patients aged <70 years, a slightly higher proportion of patients received subsequent treatment (ie, after the trial ended) with induction chemotherapy in the TC arm (21.4%) than in the decitabine arm (16.9%), and 7.1% of patients received a subsequent hypomethylating agent in the TC arm compared with no patients in the decitabine arm. In patients aged ≥70 years, a smaller proportion of patients received subsequent induction chemotherapy (6.9%, TC arm; 8.2%, decitabine arm), while 9.8% of patients in the TC arm and 2.3% in the decitabine arm subsequently received a hypomethylating agent. When analysis of OS in the age subgroups was censored for subsequent disease-modifying therapy, an HR favoring decitabine was observed for all age groups (HR = 0.73, age ≥75 years; HR = 0.80, age 70–74 years; and HR = 0.97, age <70 years).

#### Type of acute myeloid leukemia

Although AML type was not a stratification factor, the numbers of patients with de novo or secondary AML were well balanced across treatment arms, with approximately two thirds of patients having de novo AML. Response to decitabine was observed in both subgroups (Figure 2), with the greater proportion of responders in the larger de novo subgroup.

#### Baseline bone marrow count

Randomization was not stratified according to baseline bone marrow blast count, but counts were balanced between treatment arms: most patients (72%) had a blast count >30%, while 43% had a blast count >50%. Median blasts were 45.0% (range: 0–100) in the TC arm and 46.6% (range: 3–100) in the decitabine arm. For the subgroup of patients with 20% to 30% blasts, a lower proportion of patients had an ECOG PS of 0 in the decitabine arm compared with the TC arm (20% vs 31%, respectively), and a higher percentage of patients in the decitabine arm compared with the TC arm had poor-risk cytogenetics (40.0% vs 34.5%, respectively) and secondary AML (44.6% vs 39.7%, respectively). The response to decitabine was clearly demonstrated in patients with baseline bone marrow blasts >30% (Figure 2). The small subgroup of patients with 20% to 30% blasts (n = 65) demonstrated a significantly better response rate, although notable improvements were not observed in OS [8].

#### Baseline cytogenetic risk

Approximately two thirds of patients had intermediate-risk cytogenetics at baseline, including those with a normal karyotype. A trend toward an OS benefit with decitabine treatment was observed in this subgroup [8]. As expected, the subgroup of patients with baseline poor-risk cytogenetics had a shorter median OS [8] and somewhat lower response rates than those in the group with intermediate-risk cytogenetics (Figure 2).

#### Baseline ECOG performance score

Randomization was stratified for ECOG PS; approximately 74.3% of patients had ECOG PS of 0 or 1. As expected, patients with ECOG PS of 2 showed shorter median OS by treatment arm versus patients with ECOG PS of 0 or 1 [8]. Also as expected, response rates were greater in patients with ECOG PS of 0 or 1 (Figure 2). Nevertheless, the subgroup of patients with ECOG PS of 2 showed an OS benefit with decitabine treatment (HR = 0.65; p = 0.025).

#### Geographic region

**Figure 3:**
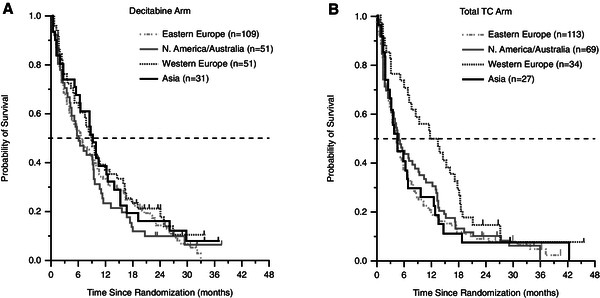
**Overall survival by treatment arm and by geographic region (2010 mature analysis data set). (A)** Decitabine arm; **(B)** Treatment choice [TC] arm.

No stratification for geographic region was considered at randomization, thus some imbalance was noted in the number of patients in each treatment arm in some regions. In particular, the Western European subgroup had more patients in the decitabine arm (n = 51) versus the TC arm (n = 34), and the North American/Australian subgroup had fewer patients in the decitabine arm (n = 51) than in the TC arm (n = 69). In the Western European subgroup, OS appeared to differ markedly from the findings in the primary study [8] (Figure 3).

Patients from the Western European region in both the decitabine and TC arms received more cycles of therapy than did patients in the other regions. In Western European patients in the TC arm, fewer patients had ECOG PS of 2, poor-risk cytogenetics, or baseline bone marrow count >50% compared with patients in the decitabine arm. Additionally, more patients in the decitabine arm were aged >75 years and had poor-risk cytogenetics, baseline bone marrow blast count >50%, or secondary AML. Unlike other regions, the Western European region showed a markedly increased use of subsequent hypomethylating agents in the TC versus decitabine arm: more than one third of patients (n = 12 [35%]) in the TC arm subsequently received treatment with a hypomethylating agent, while only 4 patients (8%) in the decitabine arm were retreated with a hypomethylating agent (including decitabine).

## Discussion

Decitabine therapy was previously associated with improved (but nonsignificant) outcomes compared with TC (cytarabine or SC) in older patients with AML [8]. This multivariate analysis of data from a large, randomized phase 3 trial in older patients with newly diagnosed AML demonstrated that all subgroups (age, type of AML, bone marrow blasts, cytogenetic risk, and ECOG PS) had responses favoring decitabine, even those with poorer prognostic factors, such as baseline bone marrow blasts >30%. Moreover, consistent with known prognostic factors for older patients with AML [10,11], patients aged ≥75 years and 70–74 years had worse prognoses compared with patients <70 years. Additionally, poorer baseline ECOG PS and disease-related factors, such as poor cytogenetics, higher bone marrow blast counts, low baseline platelet counts, and high WBC counts, all predicted worse OS. Interestingly, neither the type of AML nor patient sex influenced OS.

Despite the association between age and poorer prognosis replicated in these post hoc analyses, the overall trend for treatment benefit with decitabine was more clearly observed in patients aged 70–74 years and in patients ≥75 years compared with those <70 years. Hazard ratios for OS were 0.79 (p = 0.165) and 0.72 (p = 0.035) for patients aged 70–74 and ≥75 years, respectively [8]. Patients aged <70 years demonstrated response rates of 16.9% and median OS of 9.1 months [8], which were consistent with overall results for decitabine-treated patients.

Survival and response results in older patients reported in this trial are particularly promising given that published data indicate that response and survival in clinical trials are typically diminished as the patient’s age increases. Median survival in this trial was 8.0 months in patients aged 70–74 years and 6.3 months in those aged ≥75 years [8], which compares favorably with historical data in this older population. These favorable findings for decitabine in older patients are intriguing and may warrant further investigation to determine the underlying explanation. A retrospective analysis of 968 adults with AML in 5 Southwest Oncology Group trials revealed a median OS of 6.9 months (95% CI: 5.4–7.7) in patients aged 66–75 years, but only 3.5 months (95% CI: 1.4–6.1) in patients older than 75 years [12]. In a separate analysis of 998 patients aged ≥65 years with AML or high-risk MDS treated with intensive chemotherapy, median OS in patients aged 75 and older was only 18 weeks compared with 34 weeks in patients aged 70–74 years, and 29 weeks in patients aged 65–69 years [13]. In contrast, more recent analyses of azacitidine (n = 55) in patients with 20% to 30% bone marrow blasts versus conventional care (best SC, low-dose cytarabine, or intensive chemotherapy; n = 58) [14], suggest a survival advantage for azacitidine over conventional care of 24.5 months versus 16.0 months (p = 0.005), respectively, although the atypically high median OS associated with conventional care suggests some patient selection bias. In particular, patients in the azacitidine trial had better prognostic factors than did the high-risk patients in the present decitabine trial.

Notable in the current analyses is the outcome for patients in the Western European subgroup, who had a longer median OS compared with patients from other regions. In this small subgroup (n = 85), median OS in the TC arm (12.5 months) for the primary analysis was much longer than that in any other region and was associated with a wide confidence interval. In Western Europe, fewer patients were randomized to the TC arm than to the decitabine arm, which contributed to the greater variability observed in OS curves for the TC arm and to the wide confidence intervals for HRs. In addition, fewer patients in the TC arm had factors associated with poor prognosis compared with those in the decitabine arm. Finally, there was a marked imbalance in subsequent therapy with hypomethylating agents, as more patients in the TC arm than in the decitabine arm received subsequent disease-modifying therapy with a hypomethylating agent. Thus, the OS trend seen in this subgroup might be explained by the combination of the imbalance in baseline characteristics, the high rate of subsequent disease-modifying therapy, and the greater variability of the data owing to the small sample size.

This trial and subsequent analyses had some limitations. To enable a comparison of an IV regimen of decitabine with the standard subcutaneous cytarabine regimen, the trial utilized an open-label study design. Also, sample sizes were small in some subgroups. Nevertheless, decitabine demonstrated robust and fairly consistent results across most defined subgroups in this older population with AML, with a magnitude of effect consistent with that seen in other clinical trials of decitabine, such as the phase 2 trial reported by Cashen and colleagues (median OS, 7.7 months) [7]. The underlying reasons why some subgroups with typically poor prognoses had favorable responses to decitabine are not well understood; further investigation may be warranted to determine the explanations for these findings. The reliability of the results may lend them validity, despite the trial limitations.

## Conclusions

Older patients with AML have characteristics that may adversely affect response to conventional therapy. In this multivariate analysis, response to decitabine was associated with known prognostic factors, such as age, ECOG PS, and cytogenetics. Subgroup analyses revealed that response to decitabine compared with that of cytarabine or standard care was most clearly demonstrated in patients aged ≥75 years, a population that is traditionally difficult to treat, with a poor prognosis.

## Abbreviations

AML: Acute myeloid leukemia; CI: Confidence interval; CR: Complete response; CRp: Complete response with incomplete platelet recovery; ECOG PS: Eastern cooperative oncology group performance status; HR: Hazard ratio; IV: Intravenous; MDS: Myelodysplastic syndrome; OR: Odds ratio; OS: Overall survival; PD: Progressive disease; SC: Supportive care; TC: Treatment of choice; WBC: White blood cell.

## Competing interests

JM and HMK have received research funding from Eisai Inc. JM has served as a consultant, advisory committee member, and member of the board of directors for Eisai Inc. JD has served as a remunerated consultant for Novartis and Genzyme. FR has received research funding and honoraria from Celgene and from Eisai Inc., and has received honoraria from Johnson & Johnson. PT, MJ, and YL are employees of Eisai Inc. CA, GM, XGT, and AW declare that they have no conflicts of interest. No author received an honorarium or other form of financial support related to the development of this manuscript.

## Authors’ contributions

JM served as a principal investigator and provided study patients, data analysis and interpretation, and manuscript preparation. HK participated in the conception and design of the study, served as a principal investigator, provided study patients, collected/assembled data, and provided data analysis and interpretation. CA and AW were study investigators, provided study patients, collected and assembled data, and provided data analysis and interpretation. JD was a study investigator, provided study patients, and collected and assembled data. GM was a study investigator, provided study patients, and collected and assembled data. XGT was a study investigator, provided study patients, and provided data analysis and interpretation. FR was a study investigator. EB and MJ provided data analysis and interpretation and manuscript preparation. YL provided data analysis and interpretation. All authors provided critical review and revisions and have read and approved the final version of the manuscript.

## Pre-publication history

The pre-publication history for this paper can be accessed here:

http://www.biomedcentral.com/1471-2407/14/69/prepub
